# Syntactic priming in children with reading difficulties: evidence from abstract and lexically mediated effects

**DOI:** 10.3389/fpsyg.2026.1909533

**Published:** 2026-09-03

**Authors:** Xuemei Chen, Wenshan Dong, Jiaxin Hu, Xin Zheng, Juanhua Yang, Weiqiong Jin, Jinmian Yang, Peng Peng, Suiping Wang

**Affiliations:** 1 Philosophy and Social Science Laboratory of Reading and Development in Children and Adolescents (South China Normal University), Ministry of Education, Guangzhou, China; 2 School of Psychology, South China Normal University, Guangzhou, China; 3 Department of Psychology, Guangzhou University of Chinese Medicine, Guangzhou, China; 4 The Experimental School Affiliated to Shanghai Jiao Tong University, Shanghai, China; 5 School of Psychology and Entrepreneurship Education, Guangdong University of Finance, Guangzhou, China

**Keywords:** abstract structural priming, lexical boost, reading disabilities, structural priming, syntactic representation

## Abstract

**Introduction:**

Children with reading disabilities (RD) often exhibit sentence processing difficulties, yet the underlying source of these difficulties remains debated. One account attributes them to delayed development of syntactic representations due to phonological deficits, whereas another proposes that syntactic knowledge is largely preserved but its use is constrained by limitations in domain-general processing resources. The present study used a structural priming paradigm to examine sensitivity to abstract syntactic structure and lexical overlap in children with reading difficulties (RD).

**Methods:**

Third-grade children with RD (8−9 years old), age-matched typically developing (TD) children, and adults completed a sentence production priming task manipulating structural repetition and verb overlap. Bayesian analyses were additionally conducted to assess evidence for group differences.

**Results:**

Children with RD demonstrated reliable abstract structural priming and lexical boost effects, comparable to those observed in TD children. Bayesian analyses further supported the absence of reliable group differences between the two child groups. Both child groups showed weaker abstract structural priming than adults, whereas lexical boost effects did not differ reliably across groups.

**Discussion:**

These findings suggest that children with RD retain sensitivity to abstract syntactic structures and lexical information during implicit syntactic learning. The weaker structural priming observed in children than in adults may reflect developmental differences in the accessibility or use of syntactic representations. Overall, the findings support the view that sentence-level difficulties in RD may arise from constraints on domain-general processing resources rather than from reduced sensitivity to syntactic structure itself.

## Introduction

Reading comprehension requires not only accurate word recognition but also the integration of individual words into meaningful syntactic structures. Syntax provides the computational system that enables words to be combined according to grammatical principles. Importantly, syntactic representation plays a crucial role in the development of later reading skills and serves as a key developmental marker of reading proficiency (Cain, 2007; Tunmer et al., 1987; Tunmer and Hoover, 1992).

### Syntactic processing difficulties in children with reading disabilities

Children with reading disabilities (RD) often exhibit difficulties beyond word-level processing, including slower processing, reduced comprehension accuracy, and difficulties with complex syntactic structures (Breznitz and Leikin, 2000; Leikin and Assayag-Bouskila, 2004; Robertson and Joanisse, 2010; Rüsseler et al., 2007; Wiseheart et al., 2009). However, the origin of these sentence-level difficulties remains unclear. One possibility, often referred to as the delayed development account, proposes that early phonological and orthographic deficits constrain the acquisition of syntactic knowledge (Lyytinen et al., 2001; Wilsenach and Wijnen, 2004). An alternative view, the general processing deficit account, argues that children with RD may acquire syntactic knowledge but experience limitations in the domain-general resources required for efficient syntactic processing (Castles and Coltheart, 1993; Ramus, 2001).

In sum, the theoretical debate centers on whether lexical-level impairments in children with reading difficulties constrain the development of syntactic representations, or whether syntactic knowledge is relatively available but its use is limited by broader processing constraints. One important limitation of most previous studies that have used tasks such as lexical judgment, syntactic judgment, or sentence reading is that these tasks do not directly isolate syntactic representations, as they place demands on other linguistic representations (e.g., phonological and semantic), cognitive resources (e.g., working memory), and task strategies. As a result, these tasks may conflate syntactic representation with general processing demands, making it difficult to determine whether observed deficits reflect impaired syntactic knowledge or limitations in processing.

### Structural priming paradigm and syntactic learning patterns in typically developing children

To address this issue, the present study adopts a structural priming paradigm, which allows syntactic learning mechanisms to be examined through implicit adaptation to previously encountered structures. Structural priming refers to the tendency for speakers to reuse syntactic structures they have previously encountered (Pickering and Ferreira, 2008). For example, if participants previously read a double object (double object, DO) sentence (e.g., “The rockstar sold the undercover cop some cocaine”), they are more likely to produce another DO structure in a subsequent target sentence. Conversely, if they read a prepositional object (PO) sentence (e.g., “The rock star sold some cocaine to the undercover cop”), they are more likely to produce a PO structure (Bock, 1986). Importantly, structural priming provides an implicit measure of syntactic representation and learning (Branigan and Pickering, 2017), as it does not require explicit judgments and is less affected by task-related strategies.

Importantly, structural priming effects can be differentiated into abstract structural priming and lexical boost effects. Abstract structural priming occurs across different lexical items between prime and target, and reflects abstract syntactic representations; and lexical boost effect refers to enhanced priming when the same verb is repeated, reflecting lexically mediated processes (Hartsuiker and Kolk, 1998; Pickering and Ferreira, 2008). Abstract structural priming has been argued as a result of an automatically long-term error-based learning system, which persisted with even 10 interval sentences or additional working memory load task between prime and target; In contrast, lexical boost decayed under the same manipulation and have been suggested to rely partly on short-term activation or memory processes supporting the maintenance of repeated lexical-syntactic information (Bock and Griffin, 2000; Chang et al., 2006; Chen et al., 2022, 2025; Hartsuiker and Kolk, 1998). Although the present task involves spoken sentence production, structural priming is widely regarded as reflecting underlying syntactic representations that are also engaged during sentence comprehension, including reading. Therefore, the paradigm provides an indirect but informative window into sentence-level processing mechanisms relevant for reading comprehension.

This distinction is particularly relevant for understanding syntactic processing in children with RD. If syntactic difficulties in RD reflect delayed acquisition or reduced availability of abstract syntactic knowledge, children with RD may show weaker sensitivity to abstract structural information. However, reduced availability of abstract representations may also result in greater reliance on lexical-syntactic information, leading to enhanced lexical boost effects. Alternatively, if syntactic representations are relatively preserved but sentence-level difficulties arise from broader processing limitations, children with RD should show structural priming patterns broadly comparable to TD children. Differences between groups may nevertheless emerge depending on the cognitive demands required to access and utilize syntactic information during processing.

Developmental studies provide a useful benchmark for interpreting these patterns. Typically developing children demonstrate abstract structural priming from early childhood, suggesting that sensitivity to abstract syntactic structures emerges relatively early (Bencini and Valian, 2008; Messenger et al., 2012; Shimpi et al., 2007; Thothathiri and Snedeker, 2008a). However, lexical boost effects may continue to develop with increasing linguistic experience and cognitive resources (Peter et al., 2015; Rowland et al., 2012; van Beijsterveldt and van Hell, 2009). Therefore, comparing TD children and adults allows us to characterize the developmental maturity of abstract and lexically mediated syntactic learning mechanisms.

### Using structural priming to investigate syntactic representations and learning in children with reading disabilities

To date, only limited research has applied structural priming to investigate syntactic processing in individuals with reading difficulties. Kuerten (2017) reported that adolescents (11–16 years old) with developmental dyslexia showed slower and less accurate sentence comprehension than controls but exhibited stronger and more persistent structural priming effects. This finding provides initial evidence that individuals with reading difficulties can adapt to previously encountered syntactic structures.

However, several important questions remain unresolved. First, previous work has mainly focused on adolescents and did not examine syntactic learning patterns during childhood, when both reading ability and syntactic development are still undergoing substantial change. Second, existing studies have not directly differentiated abstract structural priming from lexical boost effects. Because these two effects are thought to reflect partially distinct aspects of syntactic adaptation, examining them separately is necessary to determine whether reading difficulties are associated with reduced sensitivity to abstract syntactic structures or altered reliance on lexical information. Therefore, the extent to which children with reading difficulties show atypical syntactic learning patterns remains unclear.

In the current study, we investigated whether children (8–9 years old in the present sample) with reading difficulties show atypical patterns of syntactic learning by using a structural priming paradigm. Specifically, we examined whether RD children exhibit reduced sensitivity to abstract syntactic structures or altered reliance on lexical-syntactic information. To address these questions, we compared third-grade children with RD, age-matched typically developing (TD) children, and adults in a sentence production task. Structural repetition and verb overlap between prime and target sentences were manipulated to assess abstract structural priming and lexical boost effects, respectively.

We aimed to investigate two research questions. First, we examined the developmental status of abstract and lexically mediated syntactic learning in TD children by comparing their priming patterns with those of adults. Based on previous developmental findings showing that abstract syntactic learning emerges relatively early, whereas some aspects of syntactic adaptation continue to develop with increasing linguistic experience, we expected TD children to demonstrate reliable abstract structural priming and lexical boost effects, although the magnitude of priming effects might differ from adults.

Second, more critically, we investigated whether children with RD exhibit atypical syntactic learning patterns compared with TD peers. If sentence-level difficulties in RD reflect delayed development of syntactic learning mechanisms, RD children should show reduced abstract structural priming and/or enhanced lexical boost effects. Alternatively, if syntactic learning mechanisms are relatively preserved but language difficulties arise from broader processing constraints, RD children should show structural priming patterns comparable to TD children in the present implicit paradigm.

## Methods

### Participants

56 children participated in the study, including 28 children with reading difficulties (17 males, mean age = 8.43 years) and 28 age-matched and typically developing children (20 males, mean age = 8.39 years). They were native Chinese speakers who speak Mandarin as their dominant language and were enrolled in the third grade of a public primary school of Guangzhou, China. Participants were selected from an initial screening sample of 377 third-grade students. The schools were located in urban districts and followed the standard national curriculum.

Children with reading difficulties were identified using a performance-based screening procedure commonly adopted in Chinese reading research (e.g., Jiang et al., 2026). Specifically, children were classified as having reading difficulties (RD) if they met the following criteria: (1) their scores of Chinese character recognition were at least 1.5 standard deviations lower than the average score of TD children in the same third grade (Jiang et al., 2026; Wang and Tao, 1996; Wen et al., 2015); (2) they had a Raven standardized score above 96 as assessed by the Raven’s Progressive Matrices (Raven et al., 1996); (3) they had normal hearing, normal or corrected-to-normal vision, and no ophthalmological or neurological abnormalities; (4) there was no reported history of attention-deficit/hyperactivity disorder (ADHD) based on available school records.

Because there is currently no universally adopted diagnostic criterion for developmental dyslexia in Chinese populations, the present study used a performance-based definition of reading difficulties rather than a clinical diagnosis of developmental dyslexia.^1^ A set of behavioral measures assessing reading-related cognitive skills (see 2.2. Behavioral Tests) was administered to further characterize group differences. We note that this performance-based criterion, although widely used in Chinese reading research, is relative to grade-level norms and may introduce sample dependency. This limitation is further discussed in the Discussion section.

Children were included on a voluntary basis with parental consent, and no participants had a history of neurological or developmental disorders as reported by school records.

To characterize developmental differences in syntactic learning patterns, we also tested 28 university students from South China Normal University (9males, mean age = 18.36 years) who participated as an adult comparison group. Adult participants were recruited through campus advertisements and received 80 RMB as compensation. All adult participants were native Mandarin speakers with normal or corrected-to-normal vision and no self-reported history of language or learning disorders.

We listed information of all group and the results of participants’ behavioral tests in Table 1. The study was approved by the ethics committee of the department of Psychology at the South China Normal University. Before the experiment, participants (or their guardian) signed an informed consent.

**Table 1:** Demographic characteristics and behavioral results of three groups of participants.

Measures	RD (*N* = 28)	TD (*N* = 28)	Adults (*N* = 28)	RD vs. TD	RD vs. adults	TD vs. adults
	Mean [95% CI]	Mean [95% CI]	Mean [95% CI]	*p*	*p*	*p*
Age	8.43 [8.24, 8.62]	8.39 [8.20, 8.58]	18.36 [18.06, 18.66]	/	/	/
Sex (male/female)	20/8	17/11	9/19	/	/	/
RPM	118.30 [115.59, 121.02]	118.66 [116.31, 121.01]	109.23 [106.11, 112.34]	0.849	/	/
Character recognition	500.56 [467.27, 533.85]	883.53 [859.47, 907.59]	/	<0.001	/	/
Digital span	6.57 [6.01, 7.13]	6.68 [6.02, 7.34]	8.50 [8.28, 8.72]	0.764	<0.001	<0.001
Reading span	3.68 [3.31, 4.04]	4.25 [3.78, 4.72]	5.36 [4.92, 5.80]	0.055	<0.001	<0.001
Morphological awareness						
Morpheme meaning interpretation	6.96 [6.36, 7.57]	8.29 [7.59, 8.98]	/	<0.01	/	/
Morpheme recognition	28.54 [27.13, 29.94]	30.14 [29.45, 30.83]	32.93 [32.83, 33.03]	0.012	<0.001	<0.001
Morpheme production	94.25 [89.19, 99.31]	99.32 [96.23, 102.41]	112.75 [110.66, 114.84]	0.046	<0.001	<0.001
Phonological awareness						
Vowel	11.96 [10.72, 13.21]	13.46 [12.89, 14.04]	14.43 [14.14, 14.72]	0.017	<0.001	0.088
Initial	14.93 [14.78, 15.08]	14.96 [14.89, 15.04]	15.00 [15.00, 15.00]	1.000	0.83	1.000
Delete	13.30 [13.05, 13.55]	13.44 [13.27, 13.62]	13.70 [13.63, 13.76]	0.253	<0.01	0.087
Rapid automatized naming tasks						
Picture naming (Mean of RT, ms)	11.40 [10.72, 12.09]	10.61 [9.81, 11.40]	7.13 [6.80, 7.46]	0.073	<0.001	<0.001
Picture naming: task1	11.97 [11.10, 12.83]	10.99 [10.00, 11.98]	7.50 [6.99, 8.00]	0.085	<0.001	<0.001
Picture naming: task2	10.83 [10.23, 11.44]	10.22 [9.51, 10.93]	6.76 [6.50, 7.03]	0.116	<0.001	<0.001
Number naming (Mean of RT, ms)	11.24 [10.31, 12.17]	9.67 [8.98, 10.36]	5.77 [5.38, 6.15]	<0.01	<0.001	<0.001
Number naming: task1	11.36 [10.28, 12.45]	9.85 [9.14, 10.57]	6.04 [5.62, 6.45]	<0.01	<0.001	<0.001
Number naming: task2	11.11 [10.18, 12.05]	9.48 [8.69, 10.28]	5.50 [5.12, 5.87]	<0.01	<0.001	<0.001

*RPM scores are age-normed standardized scores and should not be directly compared across age groups; We have also calculated Cohen’s d for the group comparisons based on the raw participant-level data, which are reported separately in Supplementary Table S1.

### Behavioral tests

To characterize individual differences in reading-related cognitive profiles, participants completed a battery of behavioral assessments measuring word-level linguistic abilities, phonological and morphological awareness, rapid naming, and working memory. These measures were used to characterize cognitive profiles of RD and TD children and to examine reading-related cognitive differences between groups. They were not intended as direct measures of syntactic learning mechanisms assessed by the structural priming task.

Reading ability. Reading ability was assessed by using Chinese character recognition test (CRM; Wang and Tao, 1996; Wen et al., 2015). The test consisted of 101 Chinese characters varying in frequency and difficulty for third-grade children. In this task, children saw one character and then were required to write down another character to make these two characters as a word. For example, they saw “单 ([dan1], means single),” they can make a two-character word like, “单身 ([dan1 shen1], means single).” This task assessed children’s ability to recognize individual characters and use them in meaningful lexical contexts. The CRM score was also used as the primary criterion for identifying children with reading difficulties (Jiang et al., 2026; Wang and Tao, 1996; Wen et al., 2015).

Phonological awareness. To estimate participants’ phonological awareness, we used three tasks, including rime generation, onset detection, and phoneme deletion (including deletion of initial, medial, or final phonemes; Li et al., 2011; Xue et al., 2013). In the rime generation task, participants were presented with a set of pinyin syllables sharing the same rime but different onsets. Based on this pattern, they were asked to generate another syllable belonging to the same category. For example, given the syllables “gāi,” “cāi,” and “sāi,” which all share the rime “āi,” participants were required to produce a new syllable, such as “wāi.” In the onset detection task, participants listened to an auditory pinyin syllable and were required to identify and produce its onset aloud. For example, for the syllable “pō,” the correct onset was “p,” which participants were instructed to pronounce aloud. Then in the phoneme deletion task, after hearing a pinyin syllable, participants were asked to delete the initial, medial, or final phoneme and then pronounce the remaining part aloud.

Morphological awareness. Previous research has identified morphological awareness as an important contributor to Chinese reading development (Tong et al., 2009). To estimate participant’s morphological awareness, we used three tasks measuring children’s ability to recognize, interpret, and manipulate morpheme-level information. The first task was the morpheme meaning interpretation task, which assessed children’s ability to infer and explain the meaning of individual morphemes within compound words (Jiang et al., 2026). In this task, participants saw a word, for example, “专利” (patent), and then they need to select the best explanation from four options, such as A: “专门的利益” (specialized interest), B: “由于发明创造而独自享有的利益” (exclusive rights or benefits derived from an invention), C: “在利益方面很擅长” (adept at pursuing interests), and D: “特定的利益” (particular interest). The task consisted of 12 multiple-choice questions. The task assessed children’s ability to interpret morpheme meanings rather than simply recognize whole-word meanings. The second task was the morpheme recognition task (McBride-Chang et al., 2003), which assessed children’s ability to identify and judge morphological relationships between words. The recognition task consisted of 33 two-choice. For each item, participants determined whether the same character in the target word and one of the two options conveys the same morphemic meaning. For example, the target word was “分散 ([fen1 san4], means scattered),” while option A was “分离 ([fen1 li2], means separate)” and option B was “分数 ([fen1 shu4], means score).” The third task was the morpheme production task (Liu and McBride-Chang, 2010), which assessed children’s ability to generate appropriate morphological combinations based on given linguistic contexts. The creation task consisted of 31 experimental questions and 6 practice questions. Based on the given question, participants generated a novel compound word that best expressed the intended meaning. For example, “What can we call a car and a table when they are put together?”

Rapid automatized’ naming tasks (RAN). Rapid automatized naming (RAN) was assessed using naming tasks requiring children to rapidly name familiar visual stimuli. We recorded participants’ naming latency for correctly naming numbers or object pictures after they were presented. Each set of materials was administered twice, and the reaction times for correctly named items were averaged and used as the measure of rapid naming performance (Denckla and Rudel, 1976; Liu et al., 2017).

Working memory. We used memory task to access participants’ digit span and reading span. For their digital spans, we adopt the backward digit span task (Wechsler, 2008): In each trial, participants viewed a number sequence presented sequentially for 1 s per item, then recalled the sequence in reverse order by typing it and received accuracy feedback. The initial sequence length was three items and increased by one following correct recall but remained unchanged after errors. The task terminated after two consecutive failures at the same sequence length. For their reading spans, we adopt the memory task proposed by Daneman and Carpenter (1980), which has been also used in Mandarin Chinese (e.g., Yang et al., 2020). In the reading task, participants read each sentence aloud, and then after completed one set of sentences, they recalled the final word of each sentence in the set. The maximum number of final words correctly recalled in any set was the reading span.

### Experimental design

The structural priming task employed a 2 × 2 factorial design, manipulating prime structure and verb overlap between prime and target sentences. Prime structure was manipulated by presenting either a double object (DO) or a prepositional object (PO) prime sentence. Verb overlap was manipulated by whether the verb was repeated between the prime and target sentences. In each trial, participants first heard a prime sentence paired with a picture and then described a target picture after hearing an auditory sentence fragment.

The production of target sentences following prime sentences with different verbs provided an index of abstract structural priming, reflecting participants’ sensitivity to abstract syntactic structures beyond repeated lexical information. In contrast, enhanced priming when the same verb was repeated between prime and target sentences was used to assess lexical boost effects, reflecting the additional contribution of lexically mediated processes.

To examine developmental and reading-related differences in syntactic learning patterns, we compared structural priming effects across three participant groups. Comparisons between adults and TD children were conducted to characterize developmental changes in syntactic learning patterns, whereas comparisons between TD and RD children were conducted to examine whether children with reading difficulties showed atypical syntactic learning patterns relative to typically developing peers. Thus, TD children served as the primary developmental baseline for evaluating both age-related differences and differences associated with reading difficulties.

### Materials

**Figure 1:**
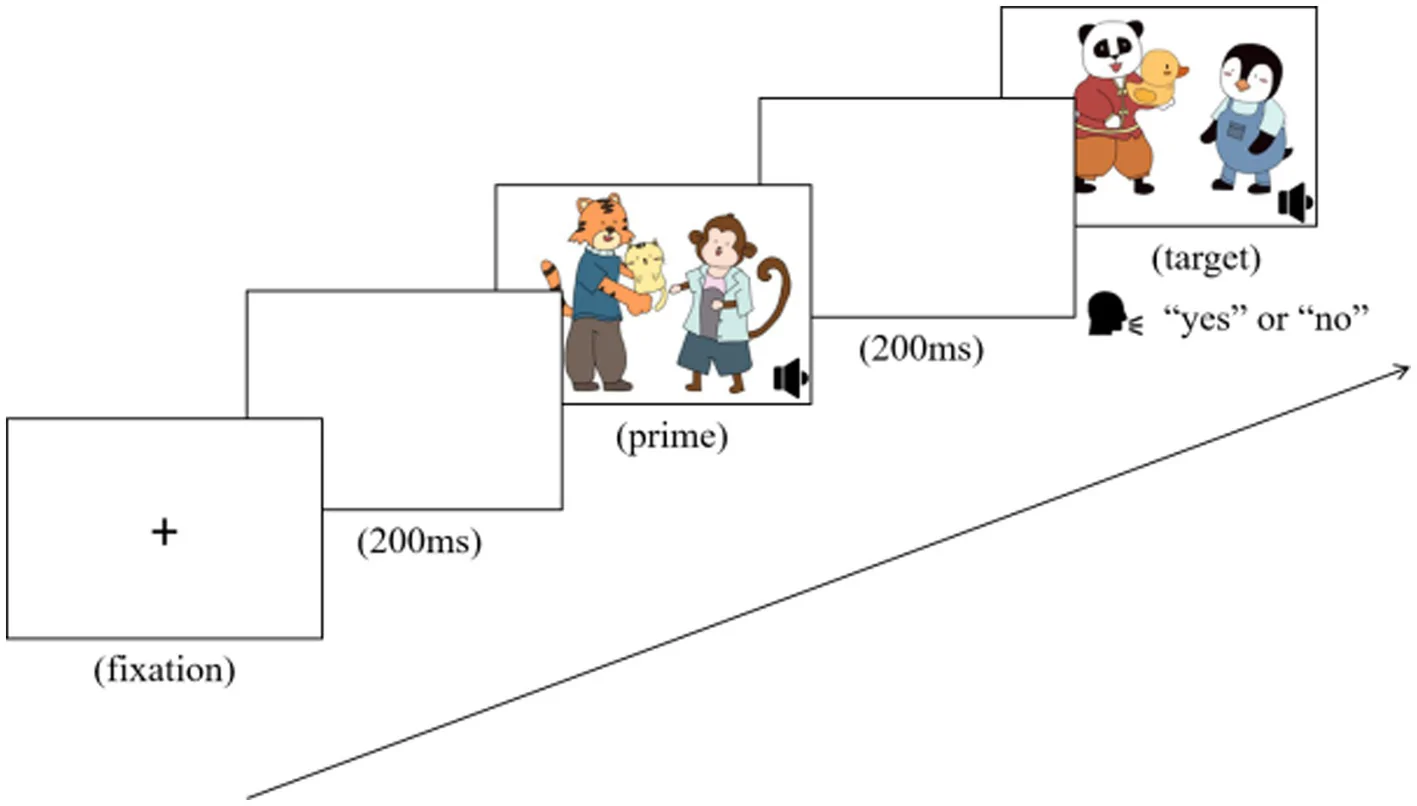
Example of one experimental trial in structural priming experiment. The prime sentence and the sentence fragment of the target picture were presented in the auditory modality.

We constructed 32 sets of experimental items. Each experimental set consisted of four prime sentences involving two structures (DO vs. PO) and two prime verbs (same vs. different verbs as target verbs, see Table 2; in auditory modality), one matching picture corresponding to the prime sentence, one target picture (see Figure 1), and one target sentence fragment (in auditory modality). The experimental design included two within-subject factors: Prime Structure (double object vs. prepositional object) and Verb Overlap (same vs. different verbs between prime and target). The DO–PO dative alternation allows for a clear comparison between two structurally distinct but semantically equivalent constructions, which was used in structural priming researches for examining syntactic representations in production (e.g., Bock, 1986; Pickering and Ferreira, 2008). Critically, the manipulation of verb overlap (same vs. different verbs) dissociates abstract structural priming from lexical boost. The different-verb priming reflects abstract representations and same-verb priming reflects additional lexical or short-term activation processes (e.g., Hartsuiker and Kolk, 1998; Branigan and Pickering, 2017).

**Table 2:** Sample stimuli in structural priming experiment.

Condition	Example
Prime sentences	
DO-same verb	老虎 带给 猴子 一只 小猫。 *(Laohu dai-gei houzi yizhi xiaomao).* *Tiger brings Monkey a Cat.*
PO-same verb	老虎 带了 小猫 给 猴子。 *(Laohu dai-le xiaomao gei houzi.)* *Tiger brings Cat to Monkey.*
DO-different verb	老虎 买给 猴子 一只 小猫。 *(Laohu mai-gei houzi yizhi xiaomao).* *Tiger buys Monkey a Cat.*
PO- different verb	老虎 买了 小猫 给 猴子。 *(Laohu mai-le xiaomao gei houzi.)* *Tiger buys Cat to Monkey.*
Target sentences	
DO	熊猫 带 给^*^ 小企鹅 一只 小鸭。 *(Xiongmao dai-gei xiaoqi’e yizhi xiaoya.)* *Panda brings penguin a duck.*
PO	熊猫 带 了^*^ 一只 小鸭 给 小企鹅。 *(Xiongmao dai-le yizhi xiaoya gei xiaoqi’e*.) *Panda brings a duck to penguin.*

Mandarin ditransitive events can be expressed using two ditransitive constructions. In the DO construction, the recipient precedes the theme (e.g., Laohu dai-gei houzi yizhi xiaomao), whereas in the PO construction, the theme precedes the recipient introduced by the preposition gei (e.g., Laohu dai-le xiaomao gei houzi). These two constructions describe the same transfer event but differ in their syntactic organization. In DO sentences, dai-gei and mai-gei function as lexical verb compounds, whereas gei in PO sentences marks the recipient. The particle le indicates event completion and was not part of the syntactic manipulation. Asterisks indicate the point at which the auditory target fragment ended, after which participants completed the sentence based on the picture.

We selected four dative verbs [i.e., “带”(bring), “买”(buy), “拿”(hand), “还”(return)] for experimental items and seven verbs (including transitive and intransitive verbs) for filler items. We selected these verbs from corpus studies of Mandarin Chinese children under 3 years old, to make sure that these verbs were learned by RD and TD group (Lee and Naigles, 2005; Ma et al., 2009).

Each prime and target picture depicted a dative event with animal and human characters as agent, recipient, and theme. This is because, according to some structural priming studies of children, priming effects were influenced by animacy feature (Gámez and Vasilyeva, 2015; Messenger et al., 2012). We counterbalanced the position of agent and recipient in these pictures (i.e., half sets of experimental items had the agent on the left and recipient on the right; The other half sets had the recipient on the left and the agent on the right); and the themes were always in the middle (Cai et al., 2011; Chen et al., 2023; Chen and Hartsuiker, 2021; Huang et al., 2019).

There were 32 sets of filler items involving transitive (i.e., SVO) and intransitive (i.e., simple SV and conjoined-subject SV) events. Filler trials were included to avoid potential carry-over effects. Each set of filler items consisted of one prime filler sentence (in auditory modality), one corresponding prime filler picture, one filler target picture, and one target sentence fragment (in auditory modality). Half filler pictures had agent on the left; and the other half had agent on the right. Similar to experimental items, all the agents, recipients, and themes were animate. Besides, to simulate the “Snap” game during structural priming paradigm in children (Branigan and McLean, 2016; Branigan and Messenger, 2016), half of fillers items had identical filler pictures between prime and target; the other half fillers had no overlap between prime and target.

We constructed 4 lists of experimental items in a Latin Square design. Each list consisted of 32 experimental trials and 32 filler trials. All trials in each list were presented in a pseudo-random order.

### Procedure

To examine implicit syntactic learning, participants completed a picture description structural priming task adapted from previous structural priming studies (Bock, 1986; Branigan and Pickering, 2017). The task was designed to examine whether exposure to previously encountered syntactic structures influenced subsequent sentence production. Specifically, participants completed the comprehension-production structural priming task (similar to Cai et al., 2011; Chen and Hartsuiker, 2021) implemented Eprime 1.0. In addition, following child-friendly structural priming paradigms such as the “Snap” game (Branigan and McLean, 2016), participants completed a picture-matching judgment task after each target description to maintain attention throughout the experiment.

Each trial consisted of a prime phase followed by a target phase. During the priming phase, participants heard the prime sentence while looking at the corresponding picture depicting a ditransitive event. The prime sentence could be a DO or PO sentence. After hearing the prime sentence, participants were presented with a target picture depicting another ditransitive event and simultaneously heard the beginning fragment of a sentence describing the target event (e.g., “熊猫带,” The panda brings). Participants were instructed to use the sentence fragment to describe the picture aloud. Their responses were recorded for subsequent coding of the produced syntactic structure (DO or PO).

After describing the target picture, participants completed a Snap judgment task, in which they judged whether the prime picture matched the target picture. If they saw the same picture in target as in prime, they spoke “shi (means yes)” aloud; otherwise, no response was required. Responses from this task were not included in the structural priming analyses. There were four practice trials before experimental phase.

### Scoring

Participants’ responses in the target description task were coded based on their produced syntactic structure. We coded a response as DO if the target verb was followed by a noun phrase indicating the recipient and then a noun phrase indicating the theme; as PO if the target verb was followed by a noun phrase indicating the theme and then a prepositional phrase indicating the preposition and the recipient. We coded DO responses as 1 and PO responses as 0 for statistical analysis. Responses that produced the alternative structure relative to the prime (e.g., a PO response following a DO prime, or vice versa) were treated as valid observations rather than errors. Structural priming effects were defined as differences in the likelihood of producing DO versus PO structures as a function of the prime condition. The sentence responses involved a non-target verb or other types of sentence structure were coded as other responses and were excluded from data analysis.

### Data analysis

Table 1 shows demographic characteristics and behavioral results of three groups of participants. In the analysis of each behavioral test, we used the “emmeans” package (Lenth et al., 2020) to compute estimated marginal means for each group from the linear model. *Post-hoc* pairwise comparisons were performed using Holm-adjusted *p*-values to control the family-wise error rate.

We used Generalized Linear Mixed Models (GLMM) with the “lme4” package in R for data analysis (Bates et al., 2015). Responses with fewer than two valid observations per condition were excluded from the analysis. We used deviation coding for three predictors: prime structure, verb overlap, and groups of participants (Chen and Hartsuiker, 2021; Scheepers et al., 2017). All predictor variables were centered to facilitate model convergence and interpretation of interaction terms. As for prime structure, we had one fixed predictor “Prime” indicating the main effect of structural priming. As for verb overlap, we had one fixed predictor “verb” indicating the main effect of verb overlap, and one fixed factor of the interaction between “Prime” and “verb” indicating lexical boost. As for three groups of participants, we had two different coding variables indicating two contrasts among three groups with the group of typically developing (TD) children serving as a baseline (see Table 3): children with reading disabilities vs. typically developing children (i.e., RD vs. TD), adults vs. typically developing children (i.e., AD vs. TD). The two-way interactions between “Prime” and one of the contrast predictors (i.e., RD vs. TD or AD vs. TD) indicated the difference of structural priming among groups of participants; And the three-way interactions among “Prime,” “verb,” and one of the contrast predictors indicated the difference of lexical boost among groups of participants. Because a model with a full maximal random effect structure did not converge (Barr, 2013; Barr et al., 2013), we included random intercepts for subjects and items, random slope of two three-way interactions for items: interaction among prime structure, verb overlap, and the contrast between RD and TD, and interaction among prime structure, verb overlap, and the contrast between adults and TD.

**Table 3:** Fixed effects for the linear mixed model.

Fixed effects	Estimate	SE	z	*p*
Prime	3.09	0.16	19.18	< 0.001
verb	0.14	0.14	0.99	0.321
RD vs. TD	−0.11	0.41	−0.27	0.788
AD vs. TD	0.06	0.40	0.15	0.882
Prime: verb	3.40	0.29	11.66	< 0.001
Prime: RD vs. TD	0.30	0.34	0.91	0.364
Prime: AD vs. TD	0.98	0.34	2.87	< 0.01
Prime:verb: RD vs. TD	−0.21	0.68	−0.31	0.760
Prime:verb: AD vs. TD	−0.02	0.69	−0.02	0.982

DO, double object construction; PO, prepositional object construction.

Given that the present study aimed to examine whether children with RD showed different syntactic learning patterns from TD children, non-significant group interaction effects were further evaluated using Bayes factors (BF) to quantify evidence supporting the null hypothesis relative to the alternative hypothesis (Lee and Wagenmakers, 2014; Wagenmakers, 2007). This approach allows us to distinguish between insufficient evidence for detecting group differences and evidence supporting the absence of meaningful group differences. Therefore, BF analyses were conducted only for theoretically relevant non-significant interaction effects in the structural priming analyses, particularly those involving group comparisons. The BF was calculated based on the difference in Bayesian information criteria (BIC) between an alternative-hypothesis linear mixed-effects model (including the relevant interaction term as a fixed effect) and a null-hypothesis model (excluding the interaction term). Larger BF values indicate stronger evidence in favor of the null hypothesis over the alternative hypothesis. Data and scripts are available online: https://osf.io/buxqk/overview.

## Results

### Results of behavioral tests

As shown in Table 1, children with reading disabilities performed worse than typically developing children in Character Recognition score, reading span, and various reading-related tests (i.e., morphological awareness, phonological awareness, picture naming, and number naming). In most of these tests, TD and RD children performed worse than adults.

### Results of structural priming experiment

Table 4 shows the frequency of target responses and priming effects (including structural priming and lexical boost) for each condition. The proportion of DO responses was calculated based on the DO and PO responses (i.e., excluding responses). Table 3 shows the results of GLMM fixed effects.

**Table 4:** Frequency of target responses for each combination of verb overlap × prime structure in three groups of participants.

Response and priming effects	RD				TD				Adults			
	Same verb		Different verb		Same verb		Different verb		Same verb		Different verb	
	DO	PO	DO	PO	DO	PO	DO	PO	DO	PO	DO	PO
do	124	12	65	34	127	16	62	32	145	7	80	26
po	97	204	150	176	88	202	155	175	76	217	138	180
other	3	8	9	14	9	6	7	17	3	0	6	18
DO%	0.56	0.06	0.30	0.16	0.59	0.07	0.29	0.15	0.66	0.03	0.37	0.13
Priming	0.51		0.14		0.52		0.13		0.62		0.24	
lexical boost	0.37				0.39				0.38			

DO, double object construction; PO, prepositional object construction.

Overall structural priming effects. The main effect of prime structure was significant (*β* = 3.09, *SE* = 0.16, 95% CI [2.78, 3.41], *z* = 19.18, *p* < 0.001), indicating a structural priming effect: participants’ target sentence productions were systematically influenced by the preceding prime structures, with a higher likelihood of producing a structure that matched the prime sentence structure. Besides, the interaction between prime structure and verb overlap was significant (*β* = 3.40, *SE* = 0.29, 95% CI [2.83, 3.97], *z* = 11.66, *p* < 0.001), indicating lexical boost effect: structural priming was stronger in the condition of same verb than that in the condition of different verbs.

Abstract structural priming: group comparisons. Critically, the interaction between prime structure and the contrast of children with reading disabilities and typically developing children (RD vs. TD) was not significant (*p* > 0.1), indicating similar effect size of structural priming between RD and TD children. The result of Bayes factor analysis showed that the null effect of interaction between prime structure and the contrast of RD vs. TD was 34 times (*BF* = 33.78) more likely than the alternative hypothesis. Additionally, the interaction between prime structure and the contrast of adults and TD was significant (*β* = 0.98, *SE* = 0.34, 95% CI [0.31, 1.65], *z* = 2.87, *p* < 0.01), indicating stronger structural priming in adults than TD children.

To further investigate whether this pattern of structural priming converging conditions of same and difference verbs among three groups of participants (i.e., a pattern characterized by stronger priming in adults than children, with no detectable difference between RD and TD children) also occurred in the condition of different verb (i.e., abstract structural priming), we then analyzed the LME model of different verb condition only. Consistently, the result showed that the interaction between prime structure and the contrast of RD vs. TD was not significant (*p* > 0.1, *BF* = 35.64), while the interaction between prime structure and the contrast of adults vs. TD was significant (*β* = 0.82, *SE* = 0.41, 95% CI [0.01, 1.63], *z* = 1.98, *p* = 0.048), indicating that abstract structural priming was similar between RD and TD children, but it was stronger in adults than in TD children.

**Figure 2:**
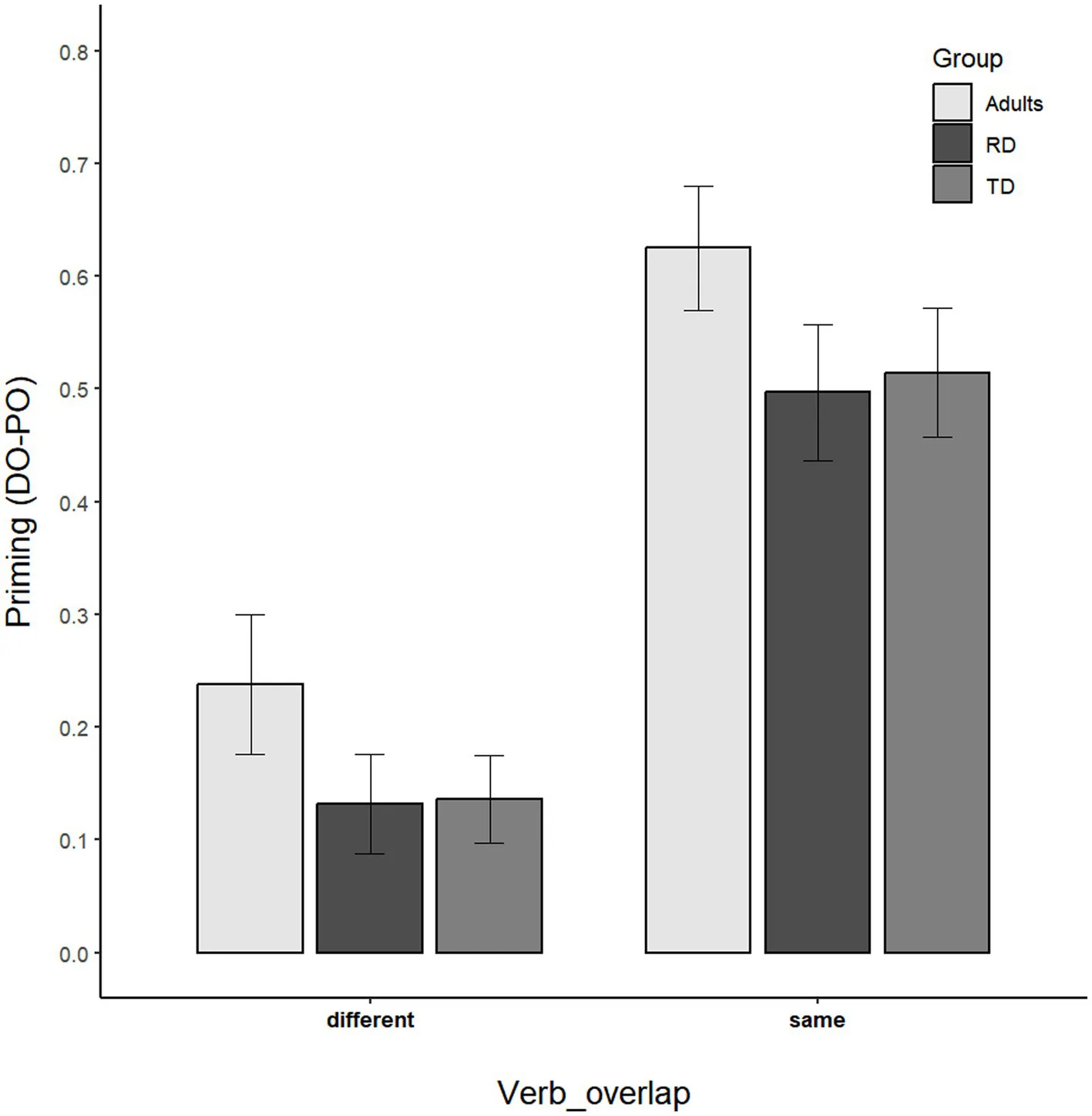
Priming effects in the groups of RD children, TD children, and adults. Prime, prime structure (including DO and PO); verb, verb overlap between prime and target (including same and different verbs conditions); RD, children with reading difficulties; TD, typically developing children; AD, adults.

Lexical boost: group comparisons. The three-way interactions among prime structure, verb overlap, and the contrast of RD vs. TD, and that among prime structure, verb overlap, and the contrast of adults vs. TD were both not significant (*p* > 0.1), indicating similar effect size of lexical boost between RD and TD children, and between adults and TD children. Furthermore, the null effect of interaction among prime structure, verb overlap and the contrast of RD vs. TD was 48 times (*BF* = 48.41) more likely than the alternative hypothesis; And the null effect of interaction among prime structure, verb overlap and the contrast of adults vs. TD was 51 times (*BF* = 50.85) more likely than the alternative hypothesis. These findings suggest that lexical boost magnitude did not differ reliably between adults and TD children, and between RD and TD children (Figure 2).

## General discussion

The present study investigated whether children with reading difficulties (RD) exhibit sensitivity to abstract syntactic structures and lexical information during implicit syntactic learning. Using a structural priming paradigm, we compared third-grade children with RD, typically developing (TD) children, and adults, while independently manipulating structural repetition and verb overlap. Two main findings emerged. First, TD children demonstrated reliable abstract structural priming and lexical boost effects, although abstract priming was weaker than that observed in adults. Second, children with RD showed structural priming patterns that were statistically indistinguishable from those of TD children, including both abstract structural priming and lexical boost effects. These findings suggest that children with reading difficulties remain sensitive to both abstract syntactic information and lexically mediated information during implicit syntactic learning. Importantly, these results should not be interpreted as demonstrating equivalent syntactic representations between RD and TD children, but rather as indicating that RD children did not show reduced sensitivity to syntactic structure under the current experimental conditions.

### Development of syntactic learning in typically developing children

The first research question examined whether third-grade (about 8–9 years old) TD children exhibit mature syntactic learning patterns. We observed robust abstract structural priming as well as reliable lexical boost effects in TD children as in adults, suggesting relatively mature syntactic representations and learning patterns in third-grade TD children. This finding is consistent with priming studies showing that abstract syntactic learning emerges early in childhood (Bencini and Valian, 2008; Branigan and McLean, 2016; Branigan and Messenger, 2016; Messenger et al., 2012; Peter et al., 2015; Rowland et al., 2012; Shimpi et al., 2007; Thothathiri and Snedeker, 2008b; van Beijsterveldt and van Hell, 2009). These findings suggest that by approximately 8–9 years of age, children have developed sufficiently abstract syntactic representations that allow them to generalize across different lexical items.

However, abstract structural priming was weaker in TD children than in adults. This pattern suggests that abstract syntactic representations are already established by the age of 8–9 years old but continue to develop in strength with increasing language experience. One possibility is that extensive language experience increases the activation strength of syntactic representations, resulting in stronger priming effects in adults. The present developmental pattern appears more compatible with activation-based accounts, such as ACT-R models (Reitter et al., 2011), which predict stronger activation of frequently encountered representations with greater experience.

At the same time, this developmental pattern differs from predictions derived from error-based implicit learning accounts (Chang et al., 2006), which propose that less experienced language users may exhibit stronger learning effects due to greater representational plasticity. The present findings therefore appear more compatible with activation-based explanations, although they do not provide a direct test between competing models. Future research manipulating linguistic experience or exposure frequency more directly will be important for determining how learning mechanisms and representational accessibility jointly contribute to developmental changes in syntactic priming.

Interestingly, lexical boost effects did not differ reliably between children and adults. This dissociation between abstract structural priming and lexical boost across age groups might suggest partially distinct mechanisms underlying these two forms of syntactic adaptation. Whereas abstract priming may reflect longer-term changes in syntactic representations, lexical boost may depend more strongly on transient activation of recently processed lexical-syntactic associations (Chen et al., 2023; Chen et al., 2025; Hartsuiker and Kolk, 1998; Malhotra et al., 2008). The absence of developmental differences in lexical boost may also reflect the highly engaging nature of the child-friendly picture description task, which may have reduced differences in task engagement between children and adults (see discussion in Branigan and McLean, 2016).

### Syntactic learning patterns in children with Reading disabilities

The most critical question concerned whether children with RD differ from TD children in syntactic learning patterns. We found that children with RD showed structural priming patterns comparable to those of TD children. Specifically, RD children demonstrated reliable abstract structural priming, suggesting that they were sensitive to structural repetition across different verbs. They also showed lexical boost effects, indicating sensitivity to the additional influence of repeated lexical information. These findings provide evidence against a strong version of the delayed development account, which predicts that reduced acquisition of syntactic knowledge should result in weaker abstract and lexically mediated syntactic learning.

Instead, the present results are more consistent with the general processing deficit account, which proposes that children with RD may possess relatively available syntactic representations but experience difficulties in efficiently applying such representations during demanding language processing. Previous studies have often reported poorer sentence comprehension and slower syntactic processing in RD children (Robertson and Joanisse, 2010; Rispens and Been, 2007). However, many of these tasks require explicit judgments, complex comprehension processes, or substantial working memory resources, making it difficult to distinguish between limitations in syntactic knowledge and limitations in processing efficiency. Structural priming provides a complementary approach by assessing implicit adaptation to syntactic structures (Branigan and Pickering, 2017). The present findings therefore suggest that sentence-level difficulties in RD may not necessarily reflect reduced sensitivity to syntactic structures themselves.

The present findings also help clarify previous inconsistent evidence from structural priming research in RD. Kuerten (2017) observed stronger and more persistent structural priming effects in adolescents with reading difficulties relative to age-matched controls. However, that study did not manipulate lexical overlap and did not directly compare abstract and lexically mediated priming effects. Moreover, stronger priming in RD may reflect differences in prior syntactic experience rather than enhanced syntactic learning ability, as predicted by implicit learning accounts (e.g., Chang et al., 2006). In contrast, the current study directly dissociated abstract structural priming from lexical boost and included age-matched TD children as the critical comparison group. The comparable priming patterns observed between RD and TD children therefore provide more direct evidence that children with reading difficulties remain sensitive to syntactic information during implicit learning.

### Lexical boost and working memory resources

An additional contribution of the present study concerns the relationship between lexical boost and domain-general cognitive resources. Previous research has suggested that lexical boost may partly arise from transient activation of recently processed lexical-syntactic information, which may depend on short-term memory resources required to maintain or retrieve repeated lexical representations (Hartsuiker and Kolk, 1998; Chen and Hartsuiker, 2023; Chen et al., 2025). Therefore, individual differences in domain-general cognitive resources may influence the extent to which speakers benefit from repeated lexical information during syntactic adaptation.

The present study found comparable lexical boost effects between RD and TD children. This finding suggests that children with RD did not show either an increased reliance on repeated lexical information or a reduced ability to benefit from lexical-syntactic repetition under the current experimental conditions. One possible explanation is that RD and TD children did not differ in digit span performance, suggesting comparable short-term memory resources available for processing and maintaining lexical-syntactic information. Thus, although broader processing limitations may contribute to sentence-level difficulties in RD, such limitations do not necessarily disrupt lexically mediated syntactic adaptation when relevant cognitive resources are sufficient.

These findings should not be interpreted as indicating that working memory does not contribute to lexical boost effects. Instead, they suggest that the influence of domain-general resources on lexical boost may depend on task demands and processing requirements. Future research should directly manipulate working memory demands during structural priming to determine whether children with RD show greater sensitivity to increased cognitive load when accessing or utilizing lexical-syntactic information. Such approaches would provide a more direct test of how domain-general processing constraints interact with syntactic learning mechanisms in children with reading difficulties.

### Limitations and future directions

Several limitations of the present study should be acknowledged. First, the sample size and sensitivity of the priming paradigm should be considered. Although the current sample size is comparable to previous structural priming studies and was sufficient to detect robust within-group priming effects (Mahowald et al., 2016), it may have limited power to detect subtle between-group differences. Moreover, the present study used a trial-based one-prime–one-target structural priming paradigm, in which priming was induced by a single preceding prime sentence. Compared with blocked priming paradigms that provide repeated exposure to the same syntactic structure, trial-based designs may produce more transient and relatively smaller priming effects. Therefore, the absence of reliable group differences should be interpreted with caution, as stronger cumulative priming effects might emerge under paradigms involving repeated structural exposure. The absence of reliable group differences in the present study should not be interpreted as strong evidence for complete equivalence, but rather suggests that any potential differences may be smaller than the current study was able to detect. Future studies with larger samples and alternative priming paradigms, including blocked designs, may provide further insights into subtle developmental differences in syntactic learning.

Second, although multiple behavioral measures were administered to characterize participants’ reading-related cognitive profiles, the classification of reading difficulties relied primarily on performance-based criteria relative to grade-level norms. While this approach is commonly used in Chinese reading research, it may introduce sample dependency and limits direct comparability with studies adopting standardized diagnostic criteria. Therefore, the findings should be interpreted as reflecting children with reading difficulties rather than clinically diagnosed developmental dyslexia. In addition, the current cross-sectional design did not allow us to determine whether these reading difficulties were persistent from earlier stages of development. Although participants were screened based on school records and cognitive assessments, we did not conduct comprehensive clinical assessments of ADHD, anxiety, or other emotional/behavioral difficulties. Therefore, the possibility of undetected comorbid conditions that may influence reading and syntactic processing cannot be completely excluded. Future research would benefit from incorporating more comprehensive and standardized diagnostic frameworks for reading disability in Chinese, including multi-component assessments of decoding, fluency, and comprehension (e.g., Shu et al., 2006; McBride-Chang et al., 2011), as well as standardized screening of developmental, attentional, and emotional/behavioral profiles. Such approaches would allow for a more fine-grained distinction between developmental dyslexia, reading delay, and broader reading difficulties, while also clarifying the contribution of domain-general cognitive factors to syntactic processing.

Third, the present study focused on a single syntactic alternation (double object vs. prepositional object constructions). Although this paradigm is well established in structural priming research (e.g., Bock, 1986; Branigan and Pickering, 2017), it remains an open question whether the observed patterns generalize to other syntactic constructions, particularly those involving greater hierarchical complexity or different argument structures.

Future research could further combine structural priming paradigms with online neurocognitive measures, such as eye-tracking or electrophysiological methods. These approaches have been shown to provide fine-grained temporal information about sentence processing and syntactic integration (e.g., Rayner, 1998; Kutas and Federmeier, 2011). The online measurements may offer valuable insights into how syntactic representations are deployed in real time. In particular, such methods may help clarify how domain-general processing constraints interact with linguistic representations during sentence comprehension and production in children with reading difficulties.

## Conclusion

The present study demonstrates that children with reading difficulties show sensitivity to both abstract syntactic structures and lexical information during implicit syntactic learning. These findings suggest that reading difficulties do not necessarily involve reduced capacity for syntactic adaptation. Instead, sentence-level difficulties in RD may arise, at least in part, from constraints on the efficient use of linguistic knowledge during processing. By distinguishing syntactic learning mechanisms from general processing limitations, the present study contributes to a more nuanced understanding of language development and reading difficulties.

## Data Availability

The datasets presented in this study can be found in online repositories. The names of the repository/repositories and accession number(s) can be found at: https://osf.io/buxqk/overview.

## Footnotes

1 Accordingly, the term reading difficulties (RD) refers to children who showed substantially lower Chinese character recognition performance relative to typically developing peers under the present screening criteria, rather than children with clinically diagnosed developmental dyslexia.
