# Supplementary material for: Syntactic priming in children with reading difficulties: evidence from abstract and lexically mediated effects
Source: Front Psychol. 2026 Sep 3;17:1909533. doi: 10.3389/fpsyg.2026.1909533 (PMC13581541; doi:10.3389/fpsyg.2026.1909533)
Supplement: Supplementary file 1 [file Table_1.DOCX]

**Supplementary Table S1**

*Effect sizes (Cohen's d) for behavioral group comparisons*

| **Measure** | **RD vs TD** | **RD vs Adults** | **TD vs Adults** |
| --- | --- | --- | --- |
| RPM | -0.06 | / | / |
| Character Recognition | -5.11 | / | / |
| Digital Span | -0.07 | -1.75 | -1.43 |
| Reading Span | -0.53 | -1.61 | -0.95 |
| Morpheme meaning interpretation | -0.79 | / | / |
| Morpheme recognition | -0.56 | -1.71 | -2.19 |
| Morpheme production | -0.47 | -1.85 | -1.97 |
| Vowel | -0.60 | -1.06 | -0.82 |
| Initial | -0.12 | -0.27 | -0.27 |
| Delete (mean) | -0.26 | -0.84 | -0.75 |
| Picture naming (RT) | 0.42 | 3.08 | 2.22 |
| Picture naming task 1 | 0.41 | 2.45 | 1.73 |
| Picture naming task 2 | 0.36 | 3.38 | 2.50 |
| Number naming (RT) | 0.74 | 2.99 | 2.70 |
| Number naming task 1 | 0.64 | 2.51 | 2.52 |
| Number naming task 2 | 0.73 | 3.05 | 2.48 |

*Note.* Cohen's d values were calculated using pooled standard deviations from raw participant-level data. Negative values indicate lower scores in the first group relative to the second group, whereas positive values indicate higher reaction times in rapid automatized naming tasks.
